# Time of Onset of Necrotizing Enterocolitis and Focal Perforation in Preterm Infants: Impact on Clinical, Surgical, and Histological Features

**DOI:** 10.3389/fped.2021.724280

**Published:** 2021-09-03

**Authors:** Janet Elizabeth Berrington, Nicholas David Embleton

**Affiliations:** ^1^Newcastle upon Tyne Hospitals National Health Service Foundation Trust, Newcastle upon Tyne, United Kingdom; ^2^Translational and Clinical Research Institute, Faculty of Medical Sciences, Newcastle University, Newcastle upon Tyne, United Kingdom; ^3^Population Health Sciences Institute, Faculty of Medical Sciences, Newcastle University, Newcastle upon Tyne, United Kingdom

**Keywords:** necrotising enterocolitis, focal perforation, timing, presentation, diagnosis, preterm infants

## Abstract

**Objective:** There is no gold standard test for diagnosis of necrotizing enterocolitis (NEC). Timing of onset is used in some definitions and studies in an attempt to separate NEC from focal intestinal perforation (FIP) with 14 days used as a cutoff. In a large, detailed data set we aimed to compare NEC and FIP in preterm infants born <32 weeks gestation, presenting before 14 days of life in comparison to cases presenting later.

**Design:** Infants with NEC or FIP when parents had consented to enrollment in an observational and sample collection study were included from 2009 to 2019. Clinical, surgical, histological, and outcome data were extracted and reviewed by each author independently.

**Patients/Episodes:** In 785 infants, 174 episodes of NEC or FIP were identified of which 73 (42%) occurred before 14 days, including 54 laparotomies and 19 episodes of medically managed NEC (“early”). There were 56 laparotomies and 45 episodes of medically managed NEC presenting on or after 14 days age (“late”).

**Results:** In early cases, 41% of laparotomies were for NEC (22 cases) and 59% for FIP (32 cases), and in late cases, 91% of laparotomies (51 cases) were for NEC and 9% (five cases) were for FIP. NEC presenting early was more likely to present with an initial septic presentation rather than discrete abdominal pathology and less likely to have clear pneumatosis. Early cases did not otherwise differ clinically, surgically, or histologically or in outcomes compared with later cases. FIP features did not differ by age at presentation.

**Conclusions:** Although most FIP occurred early, 14% occurred later, whereas almost one third (29%) of NEC cases (surgical and medical) presented early. Infant demographics and surgical and histological findings of early- and late-presenting disease did not differ, suggesting that early and late cases are not necessarily different subtypes of the same disease although a common pathway of different pathogenesis cannot be excluded. Timing of onset does not accurately distinguish NEC from FIP, and caution should be exercised in including timing of onset in diagnostic criteria.

## Introduction

Necrotizing enterocolitis (NEC) remains a devastating disease in preterm infants, and progress in prevention is slow (1–3). Preterm infants also experience other abdominal pathology, including spontaneous/focal intestinal perforation (FIP) (4). NEC and FIP are important determinants of survival and outcome in preterm infants (5–7), but they have different etiologies and preventive strategies (8–10). Enteral feeding and associated microbiome changes are classically implicated in preterm NEC (11, 12) alongside developmental dysregulation of the immune response to bacterial challenge (13, 14), whereas FIP classically occurs after minimal enteral feeding in the smallest, sickest infants and is generally considered to present at an earlier postnatal age than NEC (4, 15). In the absence of a diagnostic test or gold standard definition of NEC (16–18), age of onset has been used for FIP/NEC separation (19, 20) with recent studies using age at onset to differentiate between NEC and FIP, suggesting disease occurring before 14 days is FIP (21). Despite the potential critical importance of age at onset, accurate data on this is rare in published studies and is not included in any of the 14 NEC incidence studies (1) and only in 3/38 studies included in the meta-analysis of outcomes after NEC (3). Studies concluding that there are differences in postnatal age at NEC onset determined by birth gestation often include gestations to term or extrapolate empirical data (20, 22). However, some studies do recognize that NEC can occur early (i.e., before 14 days) with up to 40% occurring within this time frame in some preterm cohorts (23). Classification of NEC and FIP in preterm infants < 32 weeks gestation using age of onset criteria may not then be valid. We aimed to describe features at presentation, antecedent exposures, clinical (including surgical) management, and laboratory and histological findings in NEC and FIP, categorized by age at presentation, and compare disease presenting before 14 days (“early”) to “late” cases presenting thereafter. We aimed to determine if, in infants born exclusively very preterm, NEC or FIP presenting early differed from late cases, and if so, whether antecedent factors might explain these differences.

## Methods

### Cohort

Included infants were recruited to the SERVIS study (supporting enhanced research in vulnerable infants) (North East and North Tyneside 2, 10/H0908/39) (24) between 2009 and 2019 at the Royal Victoria Infirmary, Newcastle upon Tyne, United Kingdom. Following signed parental consent, this study collects data on exposures, diagnoses, management, and outcomes of infants < 32 weeks of gestation at birth along with salvaging of biological samples, such as stool, urine, and residual “waste” blood.

### Case Identification

Potential cases within SERVIS with NEC or FIP were identified through discharge letter or electronic data set (BadgerNet™) diagnosis of NEC or suspected NEC, any receipt of metronidazole, undergoing laparotomy, or dying.

Infants with potential NEC or FIP were identified, and their demographic, clinical, radiological, histological, laboratory, and outcome data (including postmortems when they occurred) extracted with all data items defined explicitly (Table 1 for key items and Supplementary Table 1 for full data set and definitions). Infants with an iatrogenic (e.g., intra-abdominal extravasation of parenteral nutrition from umbilical venous catheter) or a congenital cause for laparotomy were excluded.

**Table 1:** Key data definitions.

**Item**	**Additional data**	**Specifics/definitions**
Day full feeds		DOL first tolerated 150 mls/kg/day for 72 h
Neurodevelopment at 2 yrs CGA		Identified from BadgerNet, which classifies as normal, mild, moderate, or severe delay
Day of onset of disease	First relevant symptoms	As DOL and CGA
**Clinical presentation**		
Distension	Y/N	Recorded within 24 h of presentation
Bilious aspirates	Y/N	Recorded within 24 h of presentation
p-r bleeding	Y/N	Recorded within 24 h of presentation
Respiratory deterioration	Y/N	Requiring new pressure support (any type) or change of mode (upward)
Hypotension	Y/N	New or additional inotrope use
Overall impression at presentation	Clear abdominal focus OR sepsis like	Sepsis like = screened and treated with antibiotics but no metronidazole Clear abdominal focus = surgical opinion sought, metronidazole, transfer to surgical center
Disease location in bowel	Small bowel, large bowel, both, patchy, widespread, NEC-totalis	
Platelet count	Day 0, 1, 2, 3 Numerical	Thrombocytopenia = <100 if previously >150 or <20 below previous if previously <100 or platelet transfusion given
C-reactive protein	Day 0, 1, 2, 3	Highest value
Feed change in 72 h before NEC	New exposure to fortifier or formula, or stopping all MOM	

#### Definitions

Infants with potential NEC or FIP or who died, identified above, were reviewed independently by at least two clinicians. Surgical NEC was defined as infants who underwent laparotomy for which the combined surgical, histological, and clinical diagnoses combined were most consistent with NEC. Infants dying before laparotomy were not included. Histopathological terms were extracted from the formal histopathological report. Medical NEC was defined as receiving five or more days of antibiotics, including metronidazole and nil by mouth (NBM) with X-ray features suggestive of NEC (pneumatosis, portal venous gas, gasless, or fixed dilated loops) and no alternate diagnosis. FIP was defined as infants who underwent abdominal drainage or laparotomy or died before undergoing planned laparotomy, for which the surgical, histological, and clinical diagnoses combined were most consistent with FIP. Late onset sepsis (LOS) was defined by a positive blood culture of a single organism or mixed growth with a clinically significant organism (excluding mixed CONS species) after 72 h of age, combined with clinical features suggestive of infection, accompanied by treatment (or intention) for ≥5days with intravenous antibiotics.

LOS occurring in association with NEC was defined when infants had enteric organism–associated bacteremia while being actively managed for NEC or within 24 h of an NEC diagnosis being made.

Survival was defined as live at discharge from neonatal in-patient services and causes of death categorized as either attributable to NEC or FIP or not. Definitions were consistently applied (JB, NDE) (25).

### Disease Categorization

Episodes were classified by day of life of the first clinical feature identified as either before day 14 (early) or after this (late).

### Relevant Unit Practices

Standardized feeding regimes including promotion of and early initiation of mother's own milk (MOM), no donor milk, and preterm formula if no MOM were in use during the study. Standardized early parenteral nutrition (PN), fluconazole prophylaxis, and antibiotic regimes were used (Supplementary Material 2).

### Statistics

Data were analyzed using GraphPad Prism 8.0 and continuous data presented as median and interquartile ranges with minimum and maximum values where appropriate. Categorical data were compared by Chi-square/Fishers exact test and continuous data by multiple ANOVA/Kruskal–Wallis as appropriate.

## Results

### Episodes and Infants

Seven hundred eighty-five infants were enrolled in SERVIS during this time period, and 174 episodes were classified as NEC or FIP, excluding deaths before laparotomy (when diagnostic confirmation was lacking). Ten infants had multiple episodes of which six contributed cases to both early and late groups and seven contributed multiple episodes to the late category but always with different disease categories (i.e., NEC rather than FIP or medical rather than surgical NEC). The gestation at birth of infants with all early disease combined, early surgical or medical NEC, or early FIP did not differ from that of those presenting with all late disease or those of each type presenting later (Table 2).

**Table 2:** Clinical and laboratory features at presentation by timing and type of episode.

**Disease type**	**All NEC and FIP combined**		**NEC Surgical**		**NEC Medical**		**All NEC**		**FIP**	
Episodes*n*	<14 73	≥14 101	<14 22	≥14 51	<14 19	≥14 45	<14 41	≥14 96	<14 32	≥14 5
Gestation at birth, weeks Median (IQR)	25.9 (24.4–26.8)	26 (24.6–28)	26.1 (24.8–27.6)	26.1 (24–28)	26.28 (24.8–27.1)	26.1 (24.7–28.1)	26.1 (24.8–27.5)	26.1 (24.6–28)	24.8 (24–26.3)	24.8 (24.3–24.8)
Birthweight, g Median (IQR)	760 (640–902)	810 (640–1020)	760 (695–1065)	835 (657–1033)	860 (580–980)	800 (635–1028)	780 (662–980)	800 (645–952)	740 (632–889)	620 (590–1038)
CGA at presentation, weeks Median (IQR)	26.6 (25.4–28.1)	30 (28–32)	27.1 (25.9–29.4)	30.4 (28.2–32)	28 (15.6–30)	30.4 (28.2–32.5)	27.8 (25.9–29.4)	30.4 (28.3–32.1)	25.8 (25–27.4)	27.3 (26–28.8)
Age at presentation, days Median (IQR)	7 (5–10)	22 (16–32)	9 (5–11)	22 (16–32)	9 (6–12)	24 (16–33)	9 (6–12)	23 (16–32)	6 (3–8)	17 (16–22)
**Primary presentation** ***n*** **(%)**										
Acute abdomen	39 (54)	53 (52)	11 (50)	32 (63)	4 (21)	18 (40)	15 (39)	50 (52)	24 (75)	3 (60)
without perforation	29 (40)	51 (50)	7 (32)	31 (61)	4 (21)	18 (40)	11 (27)	49 (51)	18 (57)	2 (40)
with perforation	10 (14)	2 (2)	4 (18)	1 (2)^$^	0 (0)	0 (O)	4 (10)	1 (1)	6 (18)	1 (20)
“Sepsis-like”	33 (44)	43 (43)	11 (50)	19 (37)	**15 (79)**	**22 (49)**	**26 (63)**	**41 (43)**	7 (21)	2 (40)
PR bleeding (primary event)	4 (5)	5 (5)	1 (5)	1 (2)	2 (10)	4 (9)	3 (7)	5 (5)	1 (3)	0
**Associated features**										
Respiratory deterioration	29 (39)	65 (64)	**11 (50)**	**41 (80)**	6 (31)	23 (51)	17 (41)	54 (56)	12 (30)	1 (20)
Inotrope need	17 (23)	31 (30)	6 (27)	22 (43)	2 (10)	8 (18)	8 (19)	30 (31)	9 (27)	1 (20)
Distension	72 (97)	99 (98)	22 (100)	51 (100)	18 (95)	43 (95)	40 (98)	94 (98)	32 (97)	5 (100)
Aspirates/vomits	61 (82)	88 (87)	19 (86)	46 (90)	18 (95)	38 (84)	37 (90)	84 (87)	24 (73)	4 (80)
GI bleeding at any time	5 (7)	10 (10)	2 (9)	2 (4)	2 (10)	8 (18)	4 (10)	10 (10)	1 (3)	0
**X-ray findings:** ***n*** **(%)**										
Pneumatosis at presentation										
Unequivocal Possible Absent	18 (24) 11 (15) 55 (74)	74 (73) 10 (10) 17 (17)	**10 (45)** 2 (9) 10 (45)	**39 (76)** 3 (6) 9 (18)	**7 (37)** 8 (42) 4 (21)	**35 (77)** 7 (16) 3 (7)	**17 (41)** 10 (24) 14 (34)	**74 (77)** 10 (10 12 (12)	1 (3) 1 (3) 30 (94)	0 0 5 (100)
Gasless abdomen	5 (7)	6 (6)	0	3 (6)	1 (5)	2 (4)	1 (3)	5 (5)	4 (12)	1 (20)
Dilated loops	16 (21)	10 (10)	4 (18)	8 (15)	6 (31)	2 (4)	10 (24)	10 (10)	6 (18)	0
Free air ever	29 (39)	11 (11)	8 (36)	7 (14)	0 (0)	0 (0)	8 (20)	7 (7)	21 (64)	4 (80)
**Blood results**										
Raised CRP (>5) Day 0–3^*^n (%)	55 (74)	81 (80)	20 (91) [2^*^]	49 (96) [2^*^]	13 (68)	27 (60) [1^*^]	33 (80) [2^*^]	76 (79) [3^*^]	22 (67)	5 (100)
**Thrombocytopenic** ^**$**^										
Day 0	25 (33)	25 (25)	**11 (50)**	**13 (25)**	4 (21)	8 (17)	**15 (36)**	**22 (23)**	10 (30)	4 (80)
Day 0–3^*^	35 (47)	46 (45)	15 (68) [2^*^]	32 (58) [2^*^]	5 (26)	10 (22) [1^*^]	20 (48) [2^*^]	42 (44) [3^*^]	15 (45)	4 (80)

*Excludes n*

* *infants who died before day 3*

$ *thrombocytopenia = <100, sustained <20 below previous if originally <100, or transfused*.

*Bold values denote p < 0.05 between early and late disease: all other comparisons not statistically significant*.

### Early vs. Late Disease Type

Of 174 episodes, 73 (42%) were before 14 days and 101 (58%) later. Before 14 days, there were 54 laparotomies and 19 episodes of medically managed NEC. After this, there were 56 laparotomies and 45 episodes of medically managed NEC. Before 14 days, 41% of laparotomies were for NEC (22 cases) and 59% for FIP (32 cases). On or after 14 days, 91% of laparotomies (51 cases) were for NEC and 9% (five cases) were for FIP. Overall, 14% FIP occurred on or after day 14, and 30% surgical NEC and 30% medical NEC presented before 14 days.

### Early NEC Compared With Late NEC

Presenting features: NEC infants presenting with early or late NEC (all combined, surgical, or medical NEC) did not differ significantly in their median gestation at birth or birthweight. More early NEC (all NEC combined and medical NEC) presented with a septic-like presentation (screened and treated as possible sepsis initially rather than presentation immediately leading to a diagnosis of NEC) than late NEC (*p* = 0.039). Other features, including proportion presenting with an acute abdominal picture, primary rectal bleeding, distension, aspirates or vomits, respiratory deterioration, or inotrope need was similar for early and late disease (*p* = 0.85). Surgical NEC was more likely to present with perforation in early disease than late (*p* = 0.02), but perforation was still individually rare at presentation of NEC (only 5/137 NEC cases). Respiratory deterioration was seen more often in late surgical NEC than early (*p* = 0.01), but other clinical presentation features were not statistically different in early and late surgical NEC. Early medical NEC differed from later medical NEC with more septic-like presentations in earlier disease (79 vs. 49%).

X-ray findings: Unequivocal pneumatosis was more common in late NEC (*p* < 0.001) for medical and surgical NEC, but other X-ray features did not differ in early or late disease.

Inflammatory markers: thrombocytopenia was more common in early NEC but only significantly so on the day of onset (*p* = 0.02). CRP elevation did not differ in early and late disease overall or for any type of NEC (Table 2).

### Early FIP Compared With Late FIP

Presenting features: No clinical, X-ray or laboratory features at presentation were significantly different between early and late FIP (Table 2).

### Antecedent Events

A similar proportion of early and late NEC infants had received packed red blood cell (pRBC) transfusion at any time before NEC, and there were no differences in the proportion of early or late NEC infants (medical or surgical) who were transfused in the 24 h before onset although time from last transfusion to disease was different in early (median 2 days) and late NEC (median 5 days). Unsurprisingly, more late-presenting infants had established full feeds than early-presenting infants, and more late-presenting NEC infants were exposed to non-maternal enteral feeds, but 20% of early NEC had established full feeds, and 20% of late NEC had not. New exposure (within 72 h) to fortifier/formula only occurred in late-presenting NEC. Nineteen percent of early NEC received probiotics before NEC compared with 52% of late NEC. Antibiotic exposure on days 0–5 was not different for early and late NEC, and no antibiotic use on days 0–3 was rare (Table 3).

**Table 3:** Antecedent events.

**Disease type**	**All NEC and FIP combined**		**NEC Surgical**		**NEC Medical**		**All NEC**		**FIP**	
Episodes*n*	<14 73	≥14 101	<14 22	≥14 51	<14 19	≥14 45	<14 41	≥14 96	<14 32	≥14 5
pRBC transfusion pre *n* (%)	44 (60)	62 (62)	15 (68)	33 (65)	11 (57)	25 (55)	26 (63)	58 (60)	18 (54)	4 (80)
pRBC within 24°*n* (%)	12 (16)	12 (12)	6 (27)	8 (16)	1 (5)	3 (6)	7 (17)	11 (12)	4	1
Of which (%) routine	25	58	33	63	0	66	28	72	1	0
Days from last pBRC transfusion	2	5	2	3	4	7	2	**5**	2	6
Median (IQR)	(0–5)	(1–11)	(0–5)	(0–10)	(1–5)	(2–15)	(0–5)	**(1**–**12)**	(0–4)	(1–7)
Full feed pre *n* (%)	**9** **(12)**	**82** **(81)**	**5** **(23)**	**43** **(84)**	**4** **(21)**	**38** **(84)**	**9** **(22)**	**81** **(84)**	0	1 (20)
Mouth care only pre NEC *n* (%)	**15** **(14)**	**2** **(2)**	**5** **(22)**	**2** **(4)**	2 (10)	0	**7** **(17)**	**2** **(2)**	8 (24)	0
Feed volume pre if not full, ml/kg/dayMedian (IQR)	40 (30–60)	80 (60–123)	60 (32–75)	80 (60–120)	60 (37–100)	100 (80–131)	60 (37–85)	80 (70–123)	40 (20–60)	60 (32–113)
MOM only pre n (%)	**56(76)**	**49(48)**	14(63)	21(41)	**16(84)**	**24(53)**	**30(73)**	**45(47)**	26(79)	4(80)
New fortifier or formula exposure in 72 h before n (%)	1 (1.5)	12 (12)	**0**	**8** **(16)**	0	4 (9)	**0**	**12** **(13)**	1 (3)	0
Probiotics pre n (%)	**10** **(13)**	**51** **(50)**	**3** **(14)**	**22** **(43)**	**5** **(26)**	**28** **(62)**	**8** **(19)**	**50** **(52)**	2 (6)	1 (20)
No antibiotics day 0–3*n* (%)	1 (1.5)	2 (2)	2 (9)	1 (2)	0	2 (4)	1 (2)	3 (3)	0	0
Days on antibiotics day of life 0–5 Median (IQR)	2 (2–5)	2 (2–5)	3 (2–5)	2 (2–5)	2 (2–2)	2 (2–2)	2 (2–5)	2 (2–2)	3 (2–5)	2 (2–3)

*Bold denotes p < 0.05 early vs. late*.

### Surgical and Histological Findings and Procedures

Surgery occurred in a similar time frame from presentation for early- and late-presenting disease with no statistical differences in time to surgery as a continuous or categorical variable (early surgery defined as <72 h from presentation) between early and late NEC or early and late FIP. In NEC, similar proportions of infants were managed with a stoma for early- and late-presenting disease, but for FIP, no late-presenting infants were managed with a stoma initially compared with 63% of early-presenting infants. Disease location was not different for early and late NEC or early and late FIP, but disease location was significantly different in early NEC compared with early FIP and for late NEC compared with late FIP. Median length resected for NEC was not different in early and late disease, nor was the proportion of NEC cases in which resection of more than 20 cm or no resection for pan-enteric disease occurred. Only five episodes were classified as pan-enteric NEC with days of life of onset being 10, 14, 14, 16, and 42.

Histology was obtained whenever resection occurred. Tissue soon after presentation (within 72 h) was obtained for 73% of early and 47% of late NEC and for FIP in 75% of early- and 80% of late-presenting cases. For NEC histology obtained within 72 h, the histological findings were broadly similar, and no individual histological characteristic was significantly different between early and late disease—the same being true for FIP. Histology obtained from beyond 72 h after presentation was similar to that obtained sooner for NEC whether presenting early or late. Only two cases of FIP had histology obtained after 72 h, but findings were similar to those obtained before 72 h for early and late disease (Table 4).

**Table 4:** Surgical and histological findings.

**Disease type**	**Surgical NEC**		**FIP**	
Episodes(n)	<14 (22)	≥14 (51)	<14 (32)	≥14 (5)
**Timing of surgery**				
Within 24 h	4 (18)	8 (16)	21 (65)	3 (60)
Within 24–48 h	9 (40)	12 (23)	8 (25)	1 (20)
Within 48–72 h	4 (18)	10 (20)	1 (3.5)	0
Beyond 72 h	5 (23)	21 (41)	2 (6.5)	1 (20)
**Location of disease**				
TI only	6 (27)	15 (29)	22 (69)	3 (60)
Other small bowel	9 (41)	15 (29)	6 (19)	2 (40)
Small and large continuous	4 (18)	8 (16)	0	0
Patchy SB and LB	0	4(8)	0	0
Large bowel only	1 (5)	3 (6)	4 (12)	0
Widespread	2 (9)	6 (12)	0	0
**Resection length**				
<10 cm	7 (32)	19 (37)	29 (91)	5
10–20 cm	10 (45)	15 (30)	3 (9)	0
>20 cm (includes no resection for extensive disease)	5 (23)	17 (33)	0	0
Resection length cm median (IQR)	10.5 (5–18)	10 (4.5–21.5)	2 (0.5–5.5)	1 (1–1)
Stoma formed at first NEC surgery	18 (81)	44 (86)(1 pre-existing)	20(63)	0
Tissue for histology n (%)	20 (91)	43 (84)	26 (81)	5 (100)
**Histology within 72 h of onset n (%)**	16 (73)	24 (47)	24 (75)	4 (80)
Pneumatosis	3 (19)	9 (38)	0	0
Inflammation	11 (88)	17 (70)	4 (17)	2 (50)
Necrosis and type if present	15 (94)	22 (92)	10 (42)	1 (25)
Full thickness	12 (75)	16 (67)	9 (38)	1 (25)
Ischemic/coagulation necrosis	12 (75)	14 (64)	6 (25)	0
Hemorrhagic necrosis	8 (50)	9 (38)	6 (25)	1 (25)
Ischemia	12 (75)	18(75)	20 (83)	3 (75)
Hemorrhagic ischemia	4 (25)	8 (33)	16 (67)	1 (25)
Mucosal hyperplasia	0	1 (4)	7 (29)	2 (50)
Submucosal atrophy	1 (6)	1 (4)	5 (21)	3 (75)
**Histology beyond 72 h of onset**	4 (18)	19 (37)	2 (6)	1 (20)
Pneumatosis	0	3 (16)	0	0
Inflammation	4 (100)	15 (79)	1 (50)	1 (100)
Necrosis and type if present	2 (50)	11(58)	1 (50)	0
Full thickness	2 (50)	7 (37)	1 (50)	
Ischemic/coagulation necrosis	1 (25)	8 (42)	0	
Hemorrhagic necrosis	1 (25)	2 (10)	0	
Ischemia	2 (50)	11 (58)	0	1 (100)
Hemorrhagic ischemia	0	2 (20)	–	1 (100)
Mucosal hyperplasia	0	0	1 (50)	1 (100)
Submucosal atrophy	0	0	1 (50)	0

*TI, terminal ileum; SB, small bowel; LB, large bowel*.

*No significant difference p < 0.05 for early vs. late disease*.

### Additional Outcomes

Co-occurring LOS was not different between early- and late-presenting NEC overall or at any specific time in relation to disease onset, but for late onset FIP, more babies had already experienced LOS before disease than for early FIP. PN requirement after term CGA was rare overall and not different between early- and late-presenting NEC or FIP. Death before discharge or by 2 years CGA and neurodevelopmental outcome in survivors were also not different between early- and late-onset disease of either type (Table 5).

**Table 5:** Outcomes.

**Disease type**	**All combined**		**Surgical NEC**		**Medical NEC**		**All NEC**		**FIP**	
Infants exclusive to category(*n*)	<14 (67)	≥14 (87)	<14 (22)	≥14 (42)	<14 (16)	≥14 (38)	<14 (38)	≥14 (82)	<14 (29)	≥14 (5)
**LOS** ***n*** **(%)**										
At any time	29 (43)	30 (34)	11 (50)	18 (42)	4 (25)	8 (21)	15 (39)	26 (32)	14 (48)	4 (80)
Preceding disease	4 (6)	7 (8)	2 (9)	0	1 (6)	3 (8)	3 (8)	3 (4)	**1 (3)**	**4 (80)**
On day of disease	9 (13)	9 (10)	3 (14)	6 (14)	1 (6)	3 (8)	4 (10)	9 (11)	5 (17)	–
Pre antibiotics stopping	3 (4)	4 (5)	1 (5)	4 (10)	0	0	1 (3)	4 (5)	2 (7)	–
Pre full re-feed	9 (13)	14 (16)	2 (9)	13 (31)	1 (6)	1 (3)	3 (8)	14 (17)	6 (20)	–
After re-feeding	6 (8)	2 (2)	3 (14)	1 (2)	1 (6)	1 (3)	4 (10)	2 (2)	2 (7)	–
Days to full feeds after disease onset Median (IQR)			17 (15–32)	19 (14–21)	17 (14–24)	13 (10–16)			19 (18–24)	19 (15–24
PN post term *n* (%)	4 (6)	9 (10)	2 (9)	9 (21)	0	0	2 (5)	9 (10)	2 (7)	0
Died pre NICU d/c n (%)	13 (19)	14 (16)	6 (27)	10 (23)	1 (6)	2 (5)	7 (18)	12 (14)	6 (20)	2 (40)
Certified primary cause = NEC or FIP (*n*)^*$*^	1	5	1	5	0	0	1	5	0	0
Certified secondary cause = NEC or FIP (*n*)^*$*^	5	4	3	4	0	0	3	4	2	0
Transferred for ongoing specialist care (*n*)	1	5	1	4	1	1	0	0	0	0
Death between d/c and 2 years CGA (*n*)	1	2	0	1	1	1	1	2	0	0
Certified death primary cause = NEC or FIP (*n*)	0	0	_	0	0	0	0	0	_	_
Certified death secondary cause = NEC or FIP (*n*)	0	0	_	0	0	0	0	0	_	_
Survival to 2 years corrected age *n* (%)	54 (80)	71 (82)	16 (73)	31 (74)	14 (88)	35 (92)	30 (79)	66 (80)	21 (73)	3 (60)
**ND at 2 years** ***n*** **(%)**										
Normal^a^	16	20	3	7	6	12	9 (30)	19 (29)	7 (33)	1 (33)
Mild^b^	8	7	4	0	1	2	5 (17)	6 (9)	3 (14)	1 (33)
Moderate^c^	4	6	1	0	2	3	3 (10)	5 (7)	1 (5)	1 (33)
Severe^d^	8	4	2	5	1	3	3 (10)	4 (6)	5 (23)	0
Not available (including *too young)	17 (11*)	14 (9*)	6 (3*)	19 (10*)	4 (3*)	15 (11*)	10 (33) (6*)	14 (21) (9*)	7 (33) (5*)	0

*Bold denotes p < 0.05 between <14 and ≥14 groups, all other values not significant*.

$ *primary cause = a (<28days) or 1a (>28 days), secondary cause = b, or c (<28 days) or 1b, 1c, or 2 (>28 days)*.

*ND = neurodevelopment a <3months delay, b 3–6 months delay, c 6–12 months delay, d >12 months delay*.

## Discussion

We identified 174 episodes of NEC and FIP, 42% occurring before day 14, 56% of which was NEC, meaning 30% of all NEC occurred before day 14. Conversely, a small proportion (5%) of late disease was FIP, and only 13% of FIP occurred on day 14 or later. With the exception of a higher proportion presenting “septic-like” and less frequent unequivocal pneumatosis, early-presenting NEC was otherwise similar to late-presenting NEC and early and late FIP comparable in all parameters. This suggests both that the diseases themselves do not differ substantially whether they present early or late and that our categorization of NEC and FIP cases is robust. Even though feed exposures did differ significantly between early and late NEC, the phenotype of NEC did not differ, suggesting that significant feed exposure, or lack of it, does not alone determine the phenotype of NEC.

### Comparison to Other Studies

Other authors have explored the timing of NEC in preterm populations and identified a similar proportion occurring before 14 days. In a cohort of 858 infants <33 weeks Yee et al. identified 40% of NEC occurring before day 14 with a mean (SD) onset of 7 (3.1) days in comparison to our median of day 9 (IQR 6–12) for early disease (23). We identified a similar proportion of medical and surgical NEC in our early- and late-presenting NEC. This is in contrast to Yee et al. in whose cohort surgery was statistically more likely in early disease. This cohort was extracted from the Canadian Neonatal Network, and the authors do consider that contamination of their early group with FIP is likely, which is not the case in our data set.

Like us, existing studies also demonstrate FIP beyond 14 days: Attridge et al. (26) identified 21% of FIP occurring beyond 14 days, similar to the 13% identified here. Despite this, other studies use onset at <14 days to refine classification of an episode as FIP (21). In our cohort, disease occurring before day 14 was, in fact, more likely to be NEC than FIP. This differs from proposals by others (20) and questions the role of timing utilized in the “two out of three” rule (19) to categorize disease as NEC. Such approaches to disease classification used in some studies make comparisons of relative incidences of NEC and SIP between studies difficult. Recent higher rates of FIP than NEC have been reported in some studies, but when timing has been used for definitions, this may become self-fulfilling (21).

Phenotypic differences between early and late NEC would be important for clinical recognition of disease and potentially point to different etiologies. We identified only a more septic like presentation and less unequivocal pneumatosis in early NEC compared with later. Less definitive pneumatosis in NEC infants of lower gestation has been reported previously (27) but not specifically in relation to timing of presentation, and the gestations of our early and late NEC infants did not differ. No studies of which we are aware have previously compared the histological findings in early- or late-onset disease in preterm infants. NEC in our infants was histologically similar to that previously reported (28) in terms of consistency of classic features (>90% demonstrating necrosis and >70% described as coagulation or ischemic necrosis, and >70% demonstrating inflammation). We did not identify differences in the frequency of key histological terms used to describe NEC and FIP between early- and late-onset disease of either type.

### Strengths and Limitations

This study includes a large cohort of FIP and both medical and surgical NEC. All cases were reviewed independently by two clinicians and detailed data extracted. We use day of symptom onset for data, not the day of formal diagnosis or laparotomy, both of which may lag significantly from onset and which improves validity of our data on presenting features and antecedent events. Our NICU provides care for around 1:20 preterm surgical cases in the United Kingdom (29), and our findings are likely to be generalizable. Most (82%) infants undergoing laparotomy also had histology obtained, consistent across NEC and FIP cases. Histology was least frequently obtained in whole bowel disease, limiting knowledge about this rare but important subgroup. The use of day 14 to separate early and late disease in this study is arbitrary but based on existing use in the literature. Different findings may be seen if an alternative arbitrary age is used, and exploration of this within data sets such as ours is possible, potentially utilizing machine learning as has been done in other NEC data sets (29).

### Meaning of the Study: Implications and Future Research

Our data suggest that FIP and NEC both present within 14 days of birth, and also, both present beyond this, and timing alone should not be used to discriminate. This includes in big data sets in which such features are more likely to be used as other clinical detail is often lacking. Infants presenting with early or late disease had the same gestations and birthweight profiles, suggesting that the timing of onset is not determined by these factors. Early- and late-onset NEC shared the majority of presenting features, but X-ray (not histological) pneumatosis was seen less frequently in early-onset disease, and presentation with a clear “acute abdomen” was less likely in early disease. This may reflect both genuine differences in presentation or differences in clinicians' ability to discern these in a younger infant for which other support measures required after birth in immature infants may still be ongoing. Clinicians should be aware of this when assessing infants in the first 2 weeks after birth. NEC occurring soon after blood transfusion (transfusion-associated NEC, TANEC) (30) was not more common in either early- or late-presenting disease, suggesting that any mechanism of causation in TANEC is not dependent on postnatal maturation. Likewise, the phenotype of NEC was not different in infants who had established full feeds or had minimal feed exposure. This is perhaps surprising given the previously demonstrated importance of enteral feeding in the pathogenesis of NEC and suggests a role that is not modulated through milk volume, one supported by the ADEPT and SIFT studies that saw no change in NEC rates with earlier introduction or faster increments of enteral milk (31, 32). We suggest that future neonatal trials reporting NEC or FIP as outcomes report both and attribute them as one or the other without use of a threshold age for either disease, but taking into account (and reporting) all clinical, surgical, histological, and outcome data in order to ensure as standardized and robust classification as possible. This will allow better comparison between research studies and better validate quality improvement projects focused on NEC or FIP. In addition, more robust separation and classification of NEC and FIP would facilitate the highest level of translational learning possible. Translational research in NEC using infant or maternal data, tissue (including stool, breast milk, and others) will be misleading if infant classification is not entirely robust and may go some way to explain variation in study findings related to antecedent feeding practices (33), microbiome findings before “NEC” (11, 12) [although these are also highly individual in well infants (34)], the role of CMV in NEC (35, 36) and biomarker studies (37–39)—to quote (in relation to calprotectin as a diagnostic test for NEC), “it's not the assay, it's the definition” (40). This may become increasingly important with the development of neonatal biobanks with researchers accessing tissue, milk, stool or blood from infants labelled as NEC or FIP by remote clinicians, and where researchers themselves have no access to high level clinical data (24, 41–43).

## Data Availability Statement

The raw data supporting the conclusions of this article will be made available by the authors, without undue reservation.

## Ethics Statement

The studies involving human participants were reviewed and approved by North East and North Tyneside 2, 10/H0908/39. Written informed consent to participate in this study was provided by the participants' legal guardian/next of kin.

## Author Contributions

All authors listed have made a substantial, direct and intellectual contribution to the work, and approved it for publication.

## Conflict of Interest

The authors declare that the research was conducted in the absence of any commercial or financial relationships that could be construed as a potential conflict of interest.

## Publisher's Note

All claims expressed in this article are solely those of the authors and do not necessarily represent those of their affiliated organizations, or those of the publisher, the editors and the reviewers. Any product that may be evaluated in this article, or claim that may be made by its manufacturer, is not guaranteed or endorsed by the publisher.
