# Supplementary material for: Time of Onset of Necrotizing Enterocolitis and Focal Perforation in Preterm Infants: Impact on Clinical, Surgical, and Histological Features
Source: Front Pediatr. 2021 Sep 3;9:724280. doi: 10.3389/fped.2021.724280 (PMC8446643; doi:10.3389/fped.2021.724280)
Supplement: Supplementary file 1 [file Data_Sheet_1.pdf]

Supplementary Material

Supplementary Table 1
Data and definitions

Item	Additional data	Specifics/definitions
Sepsis	+ve blood/CSF culture with single organism or mixed including 'pathogen' and treated (or expected) ≥5 days antibiotics	For every episode:<br>Blood or CSF<br>Organism identified<br>DOL cultured
MOM receipt	Ever / never	First and last DOL received
Formula receipt	Ever / never	First and last DOL received
Fortifier receipt	Ever / never	First and last DOL received
Day full feeds	DOL first tolerated 150mls/kg/day for 72 hours	
Probiotics	Ever / never / product	First and last DOL received
Antibiotics	DOL start / stop	Every individual i.v antibiotic given, every course
Fluconazole	Ever / never	First DOL received
Respiratory support	Number of days ventilated, CPAP/hi-flow and oxygen supplementation	Total days each received
Live at discharge from NICU	Includes infants transferred to paediatrics	
Death	Age, certified cause(s)	
Palliation	Limiting of life sustaining treatment	
Laparotomy	Ever / never	DOL, surgical diagnosis
Histology	NEC or non-NEC	If non-NEC what
Neurodevelopment at 2 yrs CGA		Identified from BadgerNet which classifies as normal, mild, moderate or severe delay
Day of onset of disease	First relevant symptoms	As DOL and CGA
Clinical presentation:		
Distension	Y/N	Recorded within 24 hours of presentation
Bilious aspirates	Y/N	Recorded within 24 hours of presentation
p-r bleeding	Y/N	Recorded within 24 hours of presentation
Respiratory deterioration	Y/N	Requiring new pressure support (any type) or change of mode (upwards)
Hypotension	Y/N	New or additional inotrope use
Overall impression at presentation	Clear abdominal focus OR sepsis like	Sepsis like = screened and treated with antibiotics but no metronidazole<br>Clear abdominal focus = surgical opinion sought, metronidazole, transfer to surgical centre
Abdominal drainage (not laparotomy)	Ever / never	DOL if ever
Laparotomy	Excludes drain only	DOL if ever
Surgical diagnosis	NEC or non NEC	If non NEC what
Stoma formation	Ever / never	DOL if ever
Stoma closure		DOL

Disease location in bowel	Small bowel, large bowel, both, patchy, widespread, NEC-totalis	
Length resected		
Initial AXR findings	Pneumatosis – unequivocal, possible, or none	If no pneumatosis – other abnormality
Worst AXR	Perforation yes/no	If no, most abnormal findings
Ultrasound	Ever used	If used normal or abnormal
pRBC transfusion	Ever or never before disease	If ever, number of days before disease onset
If transfused within 24 hours of disease onset	Already symptomatic when transfused, or symptom free	Symptom free = Hb below guideline threshold only
FBC parameters:		
Platelet count	Day 0,1,2,3<br>Numerical	Thrombocytopenia = <100 if previously >150 or <20 below previous if previously <100 or platelet transfusion given
Days to platelet recovery	Numbers of days before platelet transfusion stopped	
Lymphocyte count	Day 0	
Eosinophil count	Day 0	
Monocyte count	Day 0	
Haematocrit	Day 0	
C-reactive protein	Day 0,1,2,3	Highest value
Status day 7	Ventilatory status, product dependence, live/dead	Categorical
Surgical course	Single surgery, complex repeated surgery	
Duration NBM with NEC		
Days to full feed after NEC		
Days of antibiotic treatment for NEC		
Maximum milk feed before NEC (mls/kg/day)		
Parenteral nutrition use beyond term CGA	Y/N	
Feed type before NEC	MOM only, MOM and formula or fotifier.	
Feed change in 72 hours before NEC	New exposure to fortifier or formula, or stopping all MOM	
Discharge NICU outcome	D/C home or transferred	

DOL = Day of life

Supplementary Material 2

Relevant unit practices

Feeds were initiated with MOM, including oropharyngeal colostrum use, introduced from around 2016. Where there was shortfall in MOM supply, all NICUs used a partially hydrolysed milk formula until full feeds were achieved, and no units use donor human milk. Feeding guidelines recommended that milk feeds were increased at 24mls/kg/day once initial milk tolerated. Approximately 15% of the cohort was enrolled in a feeding study randomly allocating feed rates increases to 18 - or 30mL/kg/day. TPN was used from admission and discontinued once milk feeds of 150mls/kg/day were tolerated enterally. Standardised antibiotic regimes were penicillin and gentamicin for early onset sepsis, and flucloxacillin, gentamicin and amoxicillin, or vancomycin and ceftazidime for LOS. Metronidazole was added where there were any abdominal concerns. Infants <26 weeks, or exposed to third generation cephalosporins, or <32 weeks gestation with central venous catheters, received prophylactic fluconazole on admission and twice weekly until fully fed. Infants <32 weeks were routinely given probiotics from January 2013 (Royal Victoria Infirmary (RVI), Newcastle) and shortly thereafter in the other two tertiary units caring for infants <32 weeks gestation, and referring infants surgically to the RVI, after tolerating around 30ml/kg/day of enteral milk. Infloran™ (*L. acidophilus* and *B. bifidum)* was used until July 2016 when Labinic™ *(L. acidophilus*, *B. bifidum* and *B. Longum)* was introduced.
